# Anti-inflammatory and anticancer properties of *Alcea rosea* extracts: Insights from *in vitro* and *in vivo* studies

**DOI:** 10.3389/fphar.2025.1595604

**Published:** 2025-07-09

**Authors:** Ruhban Ansar Parry, Sajad Hamid Wani, Irfan Ahmad Mir, Basharat Ahmad Bhat, Mahboob Ul Hussain, Mushtaq Ahmad Mir, Nasreena Bashir, Abdalla N. Fadul, Surender Jangra, Sharad Vats, Showkat Ahmad Ganie

**Affiliations:** ^1^ Department of Clinical Biochemistry, University of Kashmir, Srinagar, India; ^2^ Department of Bioscience & Biotechnology, Banasthali Vidyapith, Tonk, Rajasthan, India; ^3^ Department of Chemistry and Biochemistry, Sharda University, Greater Noida, India; ^4^ Department of Biotechnology, University of Kashmir, Srinagar, India; ^5^ Department of Bioresources, Government College for Women, Pulwama, Jammu and Kashmir, India; ^6^ Department of Clinical Laboratory Sciences, College of Applied Medical Sciences King Khalid University, Abha, Saudi Arabia

**Keywords:** Alcea rosea extracts, anti-inflammatory, biochemical parameters, Caspase 3, COX-2, DAPI/PI staining, histopathology, MTT assay

## Abstract

**Background:**

Inflammation plays a critical role in colon carcinogenesis by dysregulating multiple signalling pathways. Targeting these inflammatory pathways is essential for effective colorectal cancer management. This study aims to investigate how *Alcea rosea* L. extracts can prevent inflammation-related colorectal cancer both *in vitro* and *in vivo*.

**Methods:**

Anti-inflammatory assays were conducted using standard protocols. Anticancer activity was evaluated by MTT assay, while protein expression was analysed via Western blotting. Metabolite identification was performed using GC-MS analysis. *In vivo* experiments were carried out in BALB/c mice, including histopathological evaluations and biochemical assays, to assess the physiological and molecular effects of the extracts. All experimental procedures followed established scientific guidelines to ensure accuracy and reliability of the results.

**Results:**

*In vitro* assays revealed that *Alcea rosea* extracts inhibited protein denaturation, nitric oxide production, and membrane hemolysis with IC_50_ values ranging from 47.46 to 268.46 μg/mL. MTT assays demonstrated potent cytotoxicity against HCT116 (IC_50_ = 30.94 μg/mL), HT29 (IC_50_ = 46.89 μg/mL), and SW480 (IC_50_ = 63.40 μg/mL) cell lines. The extracts significantly downregulated COX-2, NFκB, and PPAR-γ protein levels and induced PARP and Caspase 3 cleavage. GC-MS analysis identified anti-inflammatory and anticancer metabolites, including kaempferol derivatives, α-Tocopherol, and phytol. *In vivo*, AR-EA and AR-Met extracts attenuated LPS-induced paw edema and restored altered biochemical parameters in mice models, highlighting the extracts’ therapeutic potential against inflammation-associated colorectal cancer.

**Conclusion:**

The findings highlight the therapeutic potential of *Alcea rosea* extracts as natural anti-inflammatory and anticancer agents, offering a promising avenue for purification of metabolites which can be utilised for the prevention and management of inflammation-associated colorectal cancer.

## 1 Introduction

Chronic inflammatory mediators have pleiotropic effects in the growth of cancer. Inflammation favours carcinogenesis, malignancy, carcinoma growth, invasion, and metastatic spread. Intrinsic and extrinsic pathways are the link between inflammation and cancer. Inflammation, which is vital for defense, involves leukocyte migration to the affected site via extravasation, releasing lysosomal contents that can cause cellular damage and increased susceptibility to lipid peroxidation. Evaluating the ability of synthetic drugs or plant extracts to prevent haemolysis in red blood cell (RBC) membranes, akin to lysosomal membranes, is crucial for assessing their anticancer and anti-inflammatory efficacy. These agents protect membranes through mechanisms such as stabilization, enzyme inhibition, and antioxidant activity. Delving into their precise protective mechanisms and exploring synergistic combinations can yield innovative therapeutic approaches (Joseph et al., 2013; Abbas et al., 2014; Haidari et al., 2023; Huang et al., 2023; Cuffaro et al., 2024). *Alcea rosea* extracts, particularly ethyl acetate (EA) and methanolic (Met) extracts, show potent activity in preventing RBC membrane haemolysis, indicating enhanced membrane stabilization. They may inhibit leukocyte membrane lysis, reducing pro-inflammatory enzyme release. This finding aligns with observations in the Malva sylvestris extract (Belkhodja et al., 2024). Important markers such as COX-2 (Cyclooxygenase-2), NFκB/P65 (Nuclear factor-κB), and PPAR-γ [Poly (ADP-ribose) polymerase] play pivotal roles in inflammatory pathways. The COX-2 enzyme is crucial for synthesizing key inflammatory mediators such as prostaglandins, prostacyclins, and thromboxane from arachidonic acid during inflammation (Vane et al., 1998). PPAR-γ binds to nuclear receptors and regulates the transcription of various pro-inflammatory genes. Targeting these markers could help manage inflammatory responses effectively. Medicinal plants are rich sources of therapeutically active metabolites, and numerous novel drugs have been derived from them, offering potential benefits in controlling inflammation. Ethyl acetate and methanolic *Alcea rosea* extracts exhibit concentration-dependent NO reduction, potentially due to unidentified NO inhibitors (Ginovyan et al., 2023). We observed a significant decrease in the protein levels of COX-2, PPAR-γ, and NFκΒ/P65 in HCT116 (Human colorectal carcinoma cell line) and HT29 (Human colorectal adenocarcinoma cell line) cells after 24 h of treatment with ethyl acetate and methanolic extracts of A. rosea, suggesting that these extracts could be of potential use for the isolation of novel metabolites with anti-inflammatory and anticancer properties. Although medicinal plants like *Curcuma longa* and *Boswellia serrata* have been extensively studied for their anti-inflammatory properties, the therapeutic potential of *Alcea rosea* remains largely unexplored despite its rich ethnobotanical history and sparse pharmacological validation, particularly in inflammation-associated colorectal cancer. This gap in scientific knowledge necessitates an in-depth evaluation of *Alcea rosea* extracts for their possible dual anti-inflammatory and anticancer activities, particularly in inflammation-associated colorectal cancer, where targeted, plant-based therapies are urgently needed. Recent reviews have emphasized the role of inflammation in CRC pathogenesis (Ma and Kroemer, 2024). Moreover, the extracts can be used to treat various diseases in which inflammation plays a significant role in the progression of diseases such as colorectal cancer (CRC). The collected *Alcea rosea* L. (Family: Malvaceae) was taxonomically validated using the Plants of the World Online (http://plantsoftheworldonline.org/) and authenticated by a qualified botanist at the University of Kashmir. A voucher specimen (Voucher No. KASH-Bot/KU/AR-604-IA) has been deposited at the University Herbarium. The extraction process yielded approximately 300 g of extract from 5.3 kg of powdered material, establishing a drug-to-extract ratio of 17.6:1. To comply with best practices in ethnopharmacology, two orthogonal fingerprinting methods were employed: GC-MS analysis and preliminary thin-layer chromatography (TLC) profiling, to ensure batch-to-batch consistency and chemical integrity. *Alcea rosea* L. belongs to Malvaceae family and is used to treat renal and uterine inflammation, gastrointestinal infections with diarrhoea and vomiting, renal and urethra infections, hepatitis, malaria, arthritis, and snake bites in folkloric medicine (Hussain et al., 2014). The plant has a variety of biological functions which include anticancer (Abdel-Salam et al., 2018), antiurolithiatic, diuretic, anti-inflammatory, hepatoprotective (Hussain et al., 2014), analgesic and antibacterial actions (Lim, 2014; Dar et al., 2017). In adherence to the Four Pillars of Best Practice in Ethnopharmacology, the present study ensured (1) proper botanical identification and authentication, (2) traceable sourcing of raw plant materials, (3) standardized extraction and quality control using multiple fingerprinting techniques, and (4) scientifically validated biological assessments with transparent reporting of methodologies.

## 2 Materials and methods

### 2.1 Plant material collection and extraction

Fresh plant material of *Alcea rosea* was collected from various geographical regions of the Kashmir Valley, India. The collected plant specimen was authenticated by Mr. Akhter Hussain, a qualified taxonomist and deposited at the Centre of Plant Taxonomy, Department of Botany, University of Kashmir, under voucher specimen number KASH-Bot/KU/AR-604-IA. The seeds and flowers of *Alcea rosea* were shade-dried separately at 30°C and pulverized to powder by utilizing an electric grinder. The powdered form (5.3 kg) was subjected to successive Soxhlet extraction at 60°C–85°C with various solvents for 72 h. The derived extracts were filtered, and the solvent was entirely removed through the use of a rotary evaporator and were stored for later use at 4°C in a refrigerator (Dar et al., 2023).

### 2.2 Study timeline and experimental design

The study was conducted in two phases: *in vitro* and *in vivo*. The *in vitro* phase focused on evaluating the anti-inflammatory and anti-colorectal cancer potential of *Alcea rosea* extracts using HCT116, HT29, and SW480 colorectal cancer cell lines. The *in vivo* phase involved an LPS-induced inflammation model in BALB/c mice.

#### 2.2.1 *In Vitro* experiments

MTT assay: Performed on HCT116, HT29, and SW480 cells (treatment duration: 48 h).

DAPI/PI staining: Conducted on HCT116 and HT29 cells (treatment: 24 h).

Western blot analysis: Conducted on HCT116 and HT29 cells (treatment: 24 h).

Protein denaturation, HRBC membrane stabilization, and NO assays: Conducted across all extracts at 100, 300, and 600 μg/mL. The 25–50 mg/kg doses used in mice correspond to human equivalent doses of approximately 2–4 mg/kg, based on body surface area conversion, ensuring translational relevance and safety.

#### 2.2.2 *In Vivo* experiments (LPS-induced inflammation model in BALB/c mice)

Extract administration: AR-EA and AR-Met at 25 mg/kg and 50 mg/kg (oral gavage).

Control and reference groups: Normal saline (Group I), LPS-only (Group II), and dexamethasone (Group VII).

Assessment time points: Behavioural monitoring (0–13 days), histopathology (end of study), and blood sample collection (day 14).

### 2.3 *In vitro* study

#### 2.3.1 Protein denaturation assay

With few adjustments, this assay was carried out in accordance with Djuichou (Djuichou Nguemnang et al., 2019). We prepared a reaction mixture through a component combination of varying concentrations of different extracts of *Alcea rosea* (100, 300, and 600 μg/mL) with 5% bovine serum albumin. The reaction mixtures were heated at 37°C in an incubator for 20 min, followed by 15 min at 70°C to induce the denaturation of protein. The absorbance of the reaction mixture was measured at 660 nm. The standard drug aspirin, an acetylsalicylic acid, was selected as the affirmative control.

#### 2.3.2 Antiproteinase assay

With slight modifications, we carried out this assay following the protocol described by Leelaprakash and Dass (Leelaprakash and Dass, 2011). The reaction mixture was prepared by mixing 0.06 mg of trypsin, 1 mL of 20 mM Tris HCl buffer (pH 7.4), and 1 mL of test samples or extracts of *Alcea rosea* at varying concentrations (100, 300, and 600 μg/mL). The reaction mixtures were heated at 37°C in an incubator. After 5 min, 0.8% casein (1 mL) was added to the mixture. This combination of reactants was heated at 37°C in an incubator for 20 min. The reaction was completed by introducing 2 mL of 70% perchloric acid to the reaction mixture. The turbid mixture produced at the conclusion underwent centrifugation, and the resulting sediment was discarded. This experiment involved quantifying the absorbance of the supernatant at 210 nm.

#### 2.3.3 HRBC membrane stabilization

This analysis was conducted in compliance with the procedure mentioned earlier (Tantary et al., 2017). Several solutions were prepared with citric acid, sodium citrate, sodium chloride, and dextrose in water at concentrations of 0.05%, 0.8%, 0.42%, and 2%, respectively. Mice blood was mixed with Alsever solution, centrifuged at 3000 rpm for 10 min, and washed with isosaline solution. The final cell volume was reconstituted to 10% v/v with isosaline. Hypotonic solution (50 mM NaCl) triggered mice red blood cell lysis. The experimental mixture included 0.50 mL of initial erythrocyte suspension in 10 mM sodium phosphate-buffered saline (pH 7.4) and 5 mL of hypotonic solution spiked with *Alcea rosea* extracts at 100, 300, and 600 μg/mL in separate test tubes. The standard drug diclofenac sodium served as a positive control.

#### 2.3.4 Nitric oxide assay

We conducted a nitric oxide assay in accordance with the protocol outlined by Boora (Boora et al., 2014) with minor alterations. Different concentrations (600, 300, and 100 μg/mL) of all five *Alcea rosea* extracts were prepared alongside gallic acid as a standard. Griess reagent was freshly prepared. Equal volumes of 0.1% N-1-naphthyl ethylene-diamine and 1% SAA in 2.5% phosphoric acid were mixed. A mixture of 10 mM sodium nitroprusside in 1× PBS (pH 7.3) with *Alcea rosea* extracts underwent 3-h incubation. Griess reagent was then added. Control samples contained only 1× PBS. The reaction mixture was pipetted into a 96-well plate, and absorbance was measured at 546 nm using an ELISA microplate reader. Gallic acid served as a positive control for nitric oxide scavenging.

#### 2.3.5 MTT assay

The MTT [3-(4,5-Dimethylthiazol-2-yl)-2,5-Diphenyltetrazolium Bromide] assay was conducted following Yang’s protocol with minor adjustments (Yang et al., 2023). Human colon cancer cell lines (HCT116, HT29, SW480) from NCCS Pune were cultured in DMEM supplemented with penicillin, FBS, and streptomycin. Optimal growth conditions were maintained in a CO_2_ incubator. *Alcea rosea* extracts were obtained via Soxhlet extraction and solubilised in DMSO. Cells were plated in 96-well plates and treated with various extract concentrations. After 48 h, MTT assay was performed, and absorbance was measured at 570 nm using an ELISA microplate reader. IC_50_ values were determined by nonlinear regression analysis using the log[inhibitor] vs. normalized response (variable slope) model with GraphPad Prism version 10.1.0 software. Extract stock solutions were prepared at 100 mg/mL in DMSO and diluted directly in culture medium to achieve a target concentration of 200 μg/mL. At this concentration, 2 µL of stock was added to 1 mL of medium, ensuring that the final DMSO concentration did not exceed 0.2% (v/v), thereby minimizing any DMSO-related cytotoxicity.

#### 2.3.6 DAPI/PI staining

HCT116 and HT29 cells were treated with AR-EA and AR-Met for 24 h. After treatment, the cells were fixed with 4% paraformaldehyde and washed twice with PBS. Nuclear staining was performed using DAPI, while propidium iodide (PI) was used to identify dead cells. Next, a Floid™ Cell Imaging System (Thermo Scientific, USA) was used to visualize the stained cells (Parry et al., 2025).

#### 2.3.7 Western blotting

For Western blotting, HCT116 and HT29 cells were seeded in 100 mm cell culture dishes at 2.2 × 106 density. After 24 h, cells at 70% confluency were treated with ethyl acetate and methanol extracts for 24 h under standard conditions. Post-treatment, cells were harvested, lysed in NP-40 buffer, and stored at −80°C. Protein quantification (30–100 μg) was done by the Bradford method, followed by SDS-PAGE (10%–12%) and transfer onto PVDF membranes. Membranes were blocked with ODYSSEY LICOR buffer, incubated with primary antibodies overnight, washed thrice, and then incubated with secondary antibodies. Signal detection utilized LI-COR equipment (Guesmi et al., 2024).

#### 2.3.8 GC-MS analysis

Agilent technologies GC systems with MassHunter Qual 10.0 (GC-7000D_MS_QQQ model) (Santa Clora, CA, USA) equipped with HP-5 MS column were used to identify chemical components in *Alcea rosea* ethyl acetate (AR-EA), and methanol (AR-Met) extracts using GC-MS analysis (Gomathi et al., 2015). High-energy electrons (70 eV) were used to ionize the sample components. Pure helium gas served as the carrier gas. The temperature was gradually raised from 50-150°C at constant addition of 5°C per minute until it reached 250°C and was kept isothermally for 10 min. In a splitless mode, 4 μL of sample (AR extracts diluted with respective solvent) was injected. The metabolites retention time (t_R_) and their mass spectra were then compared to those of previously recognized metabolites in the NIST collection (NIST17.L library, 2023).

### 2.4 *In vivo* study

#### 2.4.1 Procurement of experimental animals

BALB/c mice (25–30 g) aged 8 ± 1 weeks, were procured from the CSIR-Indian institute of integrative medicine (IIIM Jammu). Animals were well maintained at the Kashmir University animal house with regular 12-h light/dark cycle, relative humidity of 50% ± 20%, temperature of 23 ± 2°C and ventilation of 10–15 air changes/hour. Mice were properly fed with food and water and libitum. Each experimental animal group consisted of 6 BALB/c mice divided into seven groups: Group-I (normal saline only), Group-II (LPS only), Group-III (LPS + 25 mg/kg AR-EA), Group-IV (LPS + 50 mg/kg AR-EA), Group-V (LPS + 25 mg/kg AR-Met), Group-VI (LPS + 50 mg/kg AR-Met) and Group-VII (LPS + 0.5 mg/kg Dexamethasone). Effect of AR-EA and AR-Met extracts on general behaviour and safety of BALB/c mice was investigated. A single oral dose of 1500 mg/kg of AR-EA and AR-Met was administered to the experimental animals via oral gavage. Following the administration, the animals were closely monitored for potential toxic effects over the next 4 hours. After 24 h, no mortality was observed, and they remained under continuous observation for 13 days. At the end of the study, the mice were euthanized through cervical dislocation, and the weights of key organs were measured to assess any changes. All *in vivo* experiments were carried in accordance with the guidelines of *Materials and methods* of the “committee for purpose of control and supervision of experiments on animals” (CPCSEA) norms (Pandey and Sharma, 2011).

#### 2.4.2 Histopathological studies

For histopathological analysis, paw tissues from mice were preserved in 10% formalin. The fixed tissues underwent a graded alcohol series to ensure complete dehydration. Subsequently, the dehydrated tissues were embedded in paraffin blocks. Thin sections, 3–5 μm in thickness, were sliced using an automated microtome. These tissue sections were then stained with hematoxylin and eosin. Finally, the stained sections were mounted with disterene phthalate xylene (DPX) and observed under Carl Zeiss Primovert (415510-1101-000) microscope (Ali et al., 2024).

#### 2.4.3 Estimation of biochemical parameters

Blood samples were obtained from mice via the retro-orbital plexus using fine glass capillaries and collected in properly labelled red-top vacutainers. To separate the serum, the vacutainers were centrifuged at 3000 rpm. The serum from each sample was then transferred into pyrogen-free Eppendorf tubes and stored at −20°C for subsequent analysis.

#### 2.4.4 Statistical analysis

All the *in Vitro* and *in vivo* experiments were repeated three times to ensure statistical validity, and the results were presented as the mean ± standard deviation. One-way ANOVA was performed, followed by Dunnett’s post-hoc test for comparison of treatment groups versus the LPS-only group. This approach was selected to emphasize the anti-inflammatory and protective effects of extracts relative to the inflammatory control. Statistical analysis was conducted using GraphPad Prism, version 10.1.0 (316) software. Results with a p-value lower than 0.05 were deemed statistically significant.

## 3 Results

### 3.1 *In vitro* assays

#### 3.1.1 Effect of *Alcea rosea* extracts on protein denaturation

Among the five tested extracts of *Alcea rosea*, the ethyl acetate extract (AR-EA) was the most potent at suppressing protein denaturation, with an inhibition percentage of 80.72 ± 2.93 (*p* < 0.001), followed by the methanolic extract of *Alcea rosea* (AR-Met), with an inhibition percentage of 71.21 ± 3.68 (*p* < 0.001) at a concentration of 600 μg/mL. The ethanolic extract (AR-E) had an inhibitory effect of 58.93 ± 3.06 (*p* < 0.01), and the aqueous extract (AR-Aq) had an inhibitory effect of 49.41 ± 2.95 (*p* < 0.05) at 600 μg/mL. The hexane extract (AR-H) showed the least inhibition (38.51% ± 1.89%) (*p* < 0.05) at 600 μg/mL. Aspirin, used as a positive control, demonstrated maximum inhibition (87.54% ± 4.02%) (*p* < 0.001) at 600 μg/mL. AR-EA and AR-Met exhibited IC_50_ values of 220.27 ± 7.69 and 268.46 ± 9.87 μg/mL, respectively, while the IC_50_ values of the other extracts ranged between 779.57 ± 39.02 and 460.35 ± 23.14 μg/mL. Aspirin had an IC_50_ value of 83.08 ± 3.98 μg/mL (Table 1).

**TABLE 1:** Percentage inhibition on protein denaturation, Proteinase activity, HRBC membrane hemolysis and Nitric oxide inhibition exhibited by various extracts of *Alcea rosea* along with their IC_50_ values.

Protein deneturation	%Inhibition			IC_50_ value (μg/mL)
	100 μg/mL	300 μg/mL	600 μg/mL	
Hexane extract	10.97 ± 0.72^NS^	29.45 ± 1.53^NS^	38.51 ± 1.89^NS^	779.57 ± 39.02^NS^
Ethyl acetate extract	39.99 ± 2.06*	56.87 ± 3.02**	80.72 ± 2.93***	220.27 ± 7.69**
Ethanol extract	27.86 ± 1.95^NS^	39.52 ± 2.91*	58.93 ± 3.06**	460.35 ± 23.14*
Methanol extract	38.13 ± 2.14*	53.44 ± 2.49*	71.21 ± 3.68**	268.46 ± 9.87**
Aqueous extract	24.08 ± 1.26^NS^	35.05 ± 1.94^NS^	49.41 ± 2.95*	607.79 ± 31.32^NS^
Aspirin	51.37 ± 3.05*	65.48 ± 3.57***	87.54 ± 4.02***	83.08 ± 3.98***
Proteinase activity				
Hexane extract	21.02 ± 1.05^NS^	30.43 ± 1.32^NS^	42.21 ± 2.10*	779.69 ± 36.54^NS^
Ethyl acetate extract	49.40 ± 2.82*	64.60 ± 3.14**	78.65 ± 3.24***	86.38 ± 5.28***
Ethanol extract	40.38 ± 2.01*	49.57 ± 2.41*	57.30 ± 2.65*	360.96 ± 19.12*
Methanol extract	42.56 ± 2.13*	61.11 ± 2.95**	70.46 ± 3.27***	190.92 ± 8.69***
Aqueous extract	32.04 ± 1.46^NS^	40.01 ± 2.24*	51.42 ± 2.02*	561.79 ± 28.10^NS^
Aspirin	51.0 1 ± 3.14*	65.84 ± 3.49**	82.17 ± 4.12***	68.49 ± 3.73***
HRBC membrane hemolysis				
Hexane extract	25.89 ± 1.23^NS^	36.84 ± 1.95^NS^	48.97 ± 2.48*	612.75 ± 29.14^NS^
Ethyl acetate extract	50.54 ± 3.43*	69.42 ± 3.73**	81.29 ± 3.99***	47.46 ± 2.17***
Ethanol extract	46.16 ± 2.73*	56.91 ± 3.15*	63.97 ± 3.31**	169.30 ± 8.59**
Methanol extract	49.90 ± 3.52*	67.85 ± 3.88***	79.85 ± 4.09***	61.33 ± 2.84***
Aqueous extract	42.82 ± 2.17*	54.13 ± 2.69*	63.06 ± 3.10**	249.37 ± 12.43*
Diclofenac sodium	51.98 ± 3.65*	71.97 ± 3.96***	86.98 ± 4.37***	38.55 ± 1.69***
Nitric oxide inhibition				
Hexane extract	33.89 ± 1.69^NS^	42.03 ± 2.08*	53.24 ± 2.65*	513.26 ± 26.29^NS^
Ethyl acetate extract	48.19 ± 3.01*	68.84 ± 3.59**	79.89 ± 3.90***	78.20 ± 3.49***
Ethanol extract	41.17 ± 2.50*	58.24 ± 2.90*	67.13 ± 3.48**	223.25 ± 11.70*
Methanol extract	44.15 ± 3.19*	63.14 ± 3.40**	72.92 ± 3.66***	152.05 ± 5.90***
Aqueous extract	38.23 ± 1.90^NS^	45.98 ± 2.36*	59.04 ± 2.93*	386.94 ± 19.47^NS^
Gallic acid	49.94 ± 3.49*	75.97 ± 3.80**	89.41 ± 4.07***	47.79 ± 1.94***

The data were presented as means ± S.D, of three independent experiments and evaluated by one-way ANOVA, and Microsoft Excel (version 2108) and GraphPad Prism, version 10.1.0 (316). NS, non-significant.

***p < 0.001 as compared with control. Differences were considered to be statistically significant if *p* < 0.05.

#### 3.1.2 Effect of *Alcea rosea* extracts on proteinase activity

While investigating the anti-proteinase capacity of various extracts of *Alcea rosea*, it was observed that there was a marked dose-dependent inhibition of proteinase activity due to treatment with the ethyl acetate extract (AR-EA), attaining a maximum inhibition of 78.65% ± 3.24% at 600 μg/mL (Table 1). The methanol extract (AR-Met) inhibited 70.46% ± 3.27%, while the percentage inhibition of the other extracts ranged between 42.21% ± 2.10% and 57.30% ± 2.65% at the maximum concentration. Aspirin, which was used as a positive control, showed an inhibition of 81.1% ± 4.055% at 600 μg/mL. AR-EA and AR-Met exhibited very low IC_50_ values, i.e., 86.38 ± 5.28 μg/mL and 190.92 ± 8.69 μg/mL, respectively, while in the other extracts, the IC_50_ values ranged between 779.69 ± 36.54 and 360.96 ± 19.12 μg/mL. Aspirin had an IC_50_ value of 68.49 ± 3.73 μg/mL.

#### 3.1.3 Effect of *Alcea rosea* extracts on HRBC membrane stabilization

All the tested extracts of *Alcea rosea* inhibited red blood cell (RBC) membrane haemolysis in a dose-dependent manner, with the ethyl acetate extract (AR-EA) exhibiting the greatest prevention of RBC haemolysis (81.29% ± 3.99%), followed by the methanolic extract (AR-Met) (79.85% ± 4.09%) at a concentration of 600 μg/mL, while the rest of the extracts showed an inhibition ranging between 48.97% ± 2.48% and 63.97% ± 3.31% at the maximum concentration. Diclofenac sodium, which served as a positive control, showed an inhibition of 86.98% ± 4.37% at 600 μg/mL. The efficacy of the AR-EA and AR-Met extracts was also evident by their IC_50_ values, which were 47.46 ± 2.17 μg/mL in AR-EA and 61.33 ± 2.84 μg/mL in AR-Met, while in the other extracts, the IC_50_ values ranged between 612.75 ± 29.14 and 169.30 ± 8.59 μg/mL. Diclofenac sodium had the lowest IC_50_ value of 38.55 ± 1.69 μg/mL (Table 1).

#### 3.1.4 Effect of *Alcea rosea* extracts on nitric oxide (NO) production

The maximum nitrite radical scavenging potential was found for the ethyl acetate extract (AR-EA), followed by the methanol extract (AR-Met), while the hexane extract showed the least scavenging ability. The ethyl acetate extract (AR-EA) showed the greatest nitrite scavenging activity (79.89% ± 3.90%) at 600 μg/mL, followed by the methanolic extract (AR-Met), which inhibited (72.92% ± 3.66%*)* at 600 μg/mL (Table 1). The percentage of NO inhibition exhibited by the other extracts ranged between 53.24% ± 2.65% and 67.13% ± 3.48%. The gallic acid used as a positive control exhibited an inhibition of 89.41% ± 4.07% at the maximum concentration. AR-EA and AR-Met had IC_50_ values of 78.20 ± 3.49 and152.05 ± 5.90 μg/mL, respectively, while the IC_50_ values of the other extracts ranged between 513.26 ± 26.29 and 223.25 ± 11.70 μg/mL. Gallic acid had the lowest IC_50_ value of 47.79 ± 1.94 μg/mL.

#### 3.1.5 Cytotoxic activity of AR-EA and AR-Met

**FIGURE 1:**
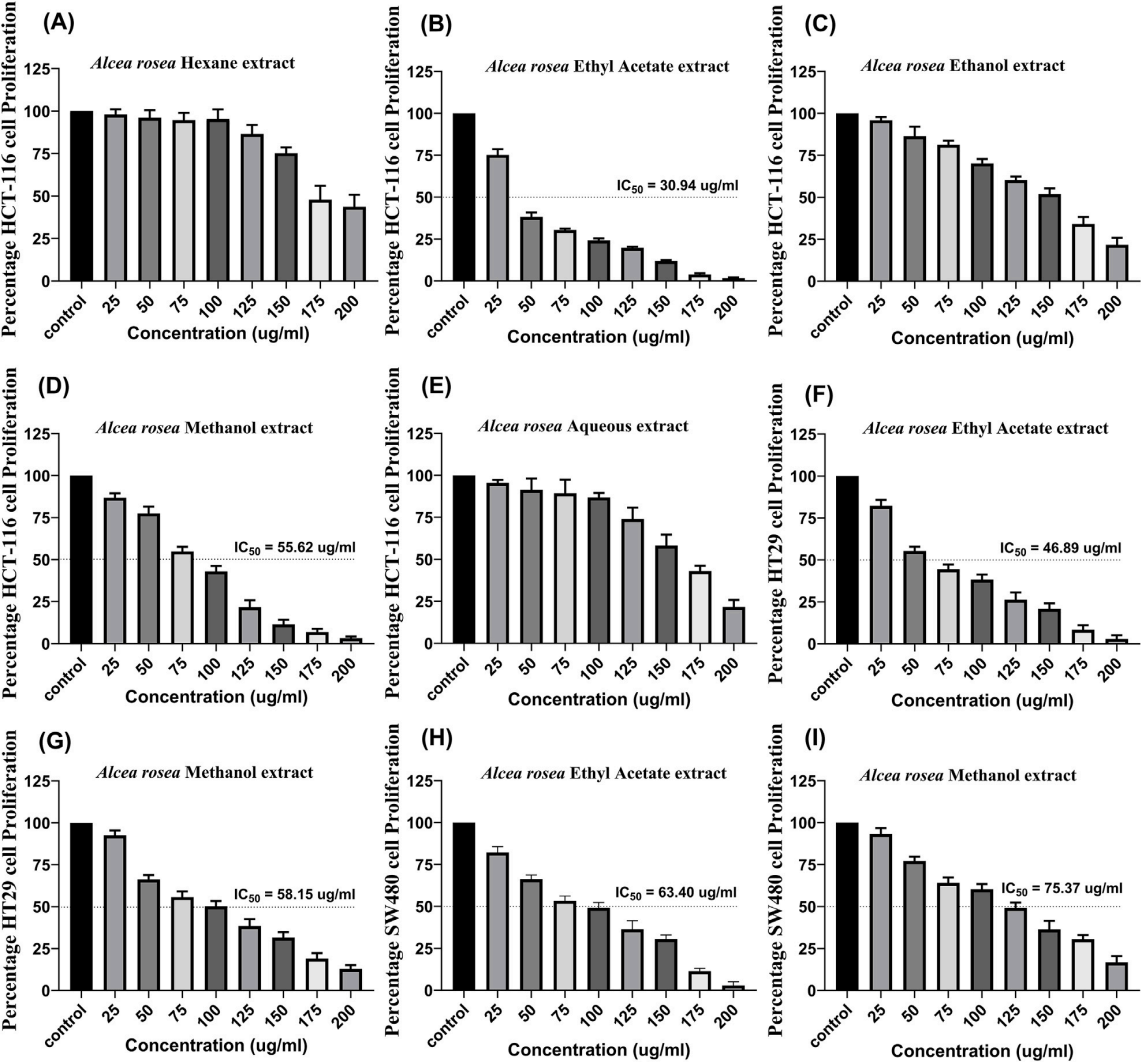
Dose-dependent effect of *Alcea rosea* extracts on the viability of colorectal cancer cell lines, along with IC_50_ values. **(A–E)** Effect of different *Alcea rosea* extracts on HCT116 cell proliferation. Ethyl acetate extract (AR-EA) showed an IC_50_ of 30.94 μg/mL, methanol extract (AR-Met) 55.62 μg/mL, followed by ethanolic extract, while aqueous and hexane extracts showed minimal effects. **(F)** Effect of AR-EA on HT29 cells (IC_50_ = 46.89 μg/mL). **(G)** Effect of AR-Met on HT29 cells (IC_50_ = 58.15 μg/mL). **(H)** Effect of AR-EA on SW480 cells (IC_50_ = 63.40 μg/mL). **(I)** Effect of AR-Met on SW480 cells (IC_50_ = 75.37 μg/mL).

MTT assay was performed on the HCT116 cell line using extracts (25, 50, 75, 100, 125, 150, 175, and 200 μg/mL). We found that *Alcea rosea* ethyl acetate extract significantly affected the viability of HCT116 cells (IC_50_ = 30.94 μg/mL), followed by *Alcea rosea* methanol extract (IC_50_ = 55.62 μg/mL) and ethanolic extract, while hexane and aqueous extracts had less inhibitory effects (Figures 1A–E). Next, we evaluated the effects of ethyl acetate extract and methanol extract on HT29 and SW480 cell lines. The ethyl acetate extract showed IC_50_ = 46.89 μg/mL in HT29 (Figure 1F) and IC_50_ = 63.40 μg/mL in SW480; Figure 1H, while the methanol extract showed IC_50_ = 58.15 μg/mL in HT29 (Figure 1G) and IC_50_ = 75.37 μg/mL in SW480; (Figure 1I).

#### 3.1.6 AR-EA and AR-Met inhibit protein levels of COX-2, NFκB and PPARγ

**FIGURE 2:**
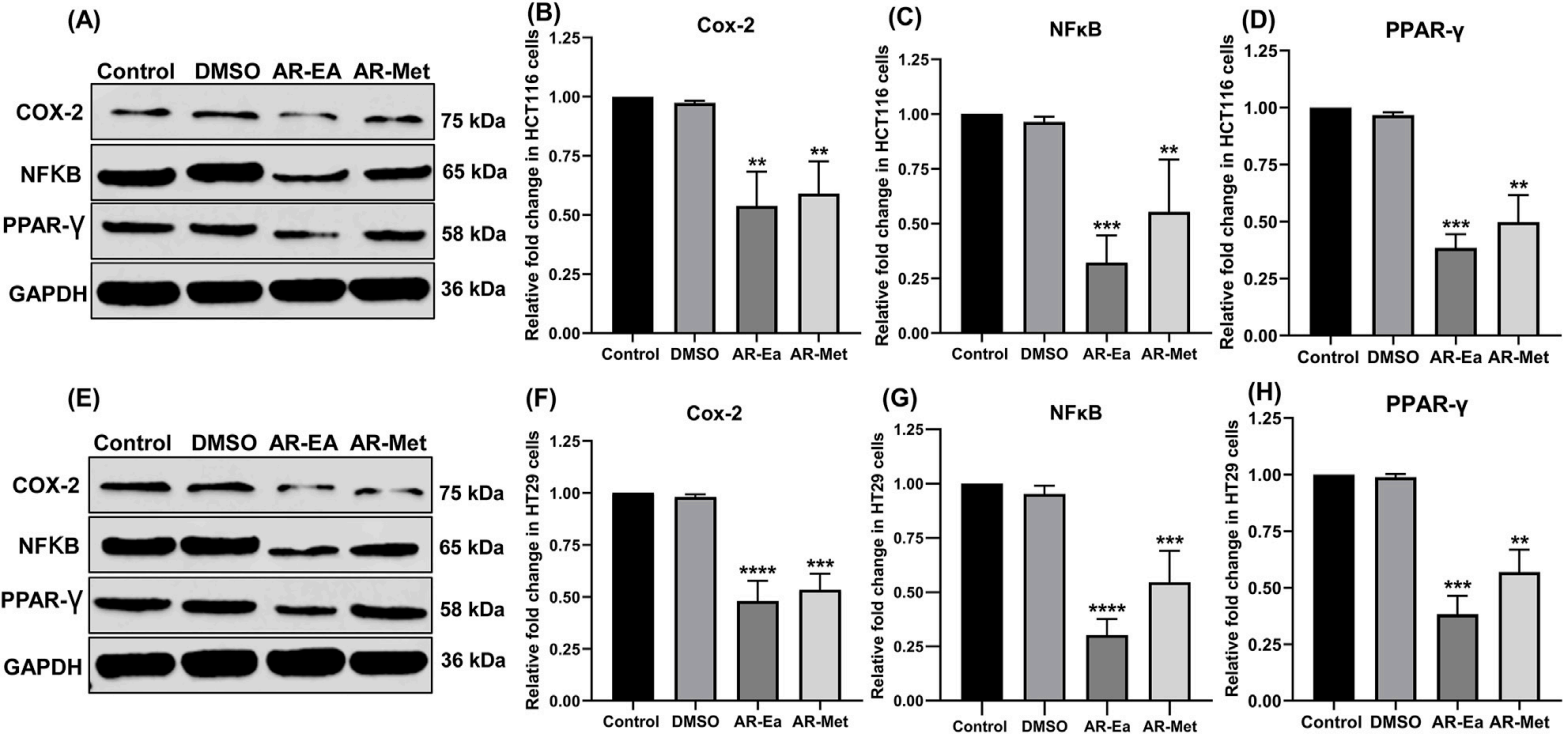
**(A)** Expression of COX-2, NFκB and PPAR-γ in ethyl acetate and methanolic extract treated HCT116 cells: Lane-1 (control); Lane-2 (DMSO); Lane-3 (AR-EA); Lane-4 (AR-Met). **(B)** Relative fold change in COX-2in HCT116 cells. **(C)** Relative fold change in NFκB expression in HCT116 cells. **(D)** Relative fold change in PPAR-γ in HCT116 cells. **(E)** Expression of COX-2, NFκB and PPAR-γ in ethyl acetate and methanolic extract treated HT29 cells: Lane-1 (control); Lane-2 (DMSO); Lane-3 (AR-EA); Lane-4 (AR-Met). **(F)** Relative fold change in COX-2 in HT29 cells. **(G)** Relative fold change in NFκB expression in HT29 cells. **(H)** Relative fold change in PPAR-γ in HT29 cells. GAPDH used as loading control. Full-length blots with molecular weight markers are provided in Supplementary Figure 1Supplementary Figure S1.

We next investigated the effect of these two extracts on key inflammatory proteins, such as COX-2, NFκB, and PPARγ, in HCT116 and HT29 cells. As shown in Figures 2A,E, the protein levels of all the probed genes decreased in both cell lines upon treatment with AR-EA and AR-Met. In HCT116 cells, AR-EA treatment decreased COX-2 levels by 1.86-fold (Figure 2B), NFκB by 3.12-fold (Figure 2C), and PPARγ by 2.6-fold (Figure 2D), and AR-Met treatment decreased COX-2 levels by 1.7-fold (Figure 2B), NFκB by 1.81-fold (Figure 2C), and PPARγ by 2.00-fold (Figure 2D). However, in HT29 cells, AR-EA treatment decreased COX-2 expression by 2.08-fold (Figure 2F), NFκB by 3.32-fold (Figure 2G), and PPARγ by 2.62-fold (Figure 2H), and AR-Met treatment decreased COX-2 expression by 1.88-fold (Figure 2F), NFκB by 1.83-fold (Figure 2G), and PPARγ by 1.76-fold (Figure 2H).

#### 3.1.7 Apoptotic activity of AR-EA and AR-Met in colorectal cancer cell lines

**FIGURE 3:**
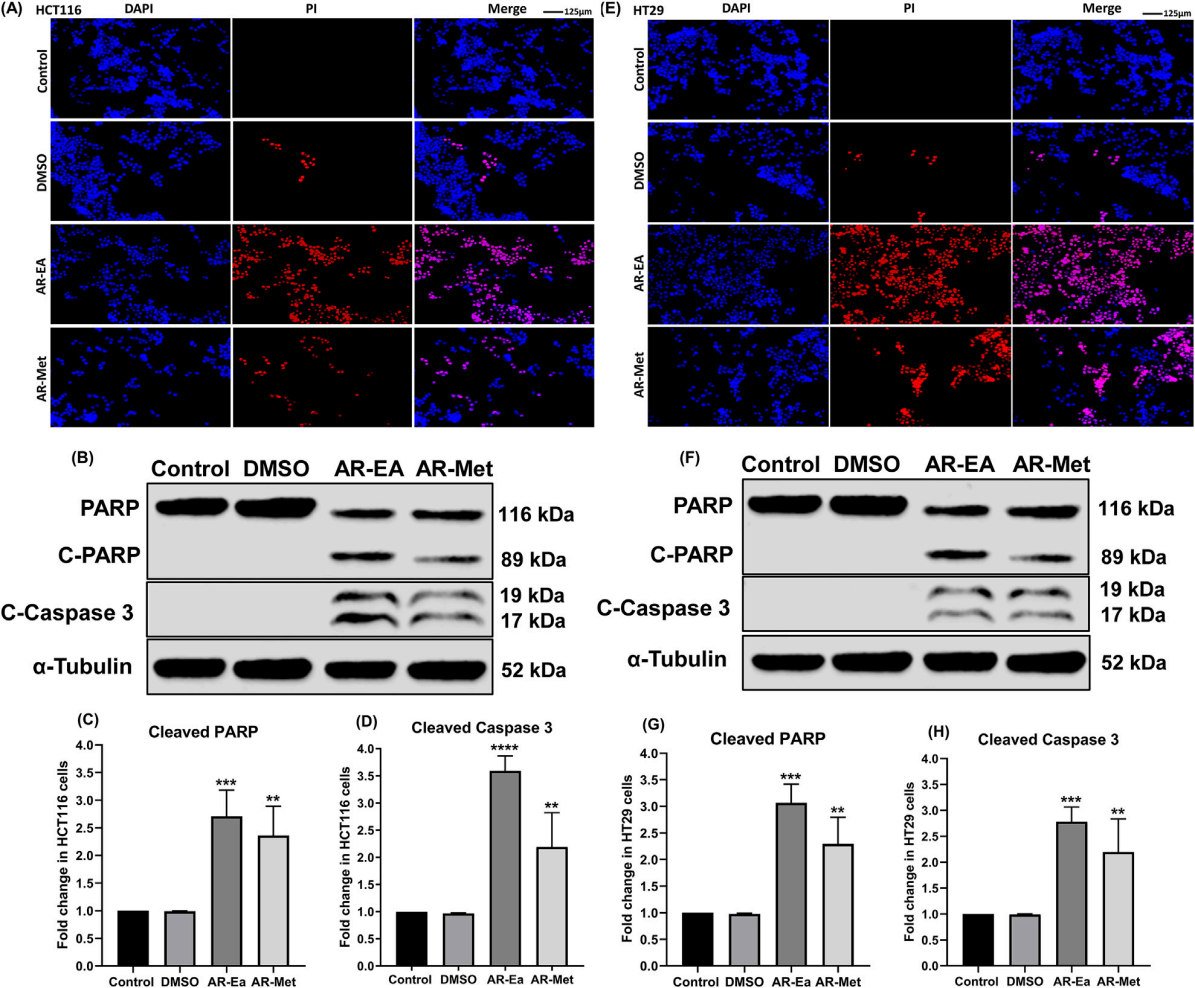
**(A)** DAPI-PI of AR-EA and AR-Met in HCT116 cells. **(B)** Expression of C-PARP and C-Caspase 3 in ethyl acetate (AR-EA)- and methanolic extract treated HCT116 cells: Lane-1 (control); Lane-2 (DMSO); Lane-3 (AR-EA); Lane-4 (AR-Met). **(C)** Fold change in C-PARP in HCT116 cells. **(D)** Fold change in the level of cleaved caspase 3 in HCT116 cells. **(E)** DAPI-PI of AR-EA and AR-Met in HT29 cells. **(F)** Expression of C-PARP and C-Caspase 3 in ethyl acetate and methanolic extract treated HT29 cells: Lane-1 (control); Lane-2 (DMSO); Lane-3 (AR-EA); Lane-4 (AR-Met). **(G)** Fold change in C-PARP in HT29 cells. **(H)** Fold change in the level of cleaved caspase 3 in HT29 cells. α-Tubulin used as loading control.

We next investigated the effect of AR-EA and AR-Met on the induction of apoptosis in HCT116 and HT29 cells. For this purpose, we exposed HCT116 and HT29 cells to AR-EA and AR-Met for 24 h. After treatment, we probed the induction of cell death in the cell lines by using DAPI(4′,6-diamidino-2-phenylindole)-PI staining, as shown in (Figure 3A) HCT116 and (Figure 3E) HT29. The exposure of HCT116 and HT29 cells to AR-EA and AR-Met for 24 h led to a significant increase in the number of apoptotic cells. These results were further validated by probing the extent of PARP and Caspase 3 cleavage. As shown in (Figures 3B,F), the protein levels of cleaved PARP and cleaved Caspase 3 were significantly increased in both cell lines upon treatment with AR-EA and AR-Met, respectively. In HCT116 cells, AR-EA treatment increased C-PARP levels 2.70-fold (Figure 3C) and C-Caspase 3 levels 3.59-fold (Figure 3D) and AR-Met treatment increased C-PARP levels 2.36-fold (Figure 3C) and C-Caspase 3 levels 2.19-fold (Figure 3D). However, in HT29 cells, AR-EA treatment increased C-PARP levels 3.06-fold (Figure 3G), C-Caspase 3 treatment 2.77-fold (Figure 3H), and AR-Met treatment 2.29-fold (Figure 3G) and C-Caspase 3 treatment 2.29-fold (Figure 3H). It can be inferred from these experimental findings that the AR-EA and AR-Met extracts of *A. rosea* display strong anticancer potential in HCT116 and HT29 colorectal cancer cell lines by mediating PARP and Caspase 3 cleavage.

#### 3.1.8 GC-MS analysis of AR-EA and AR-Met extracts

**FIGURE 4:**
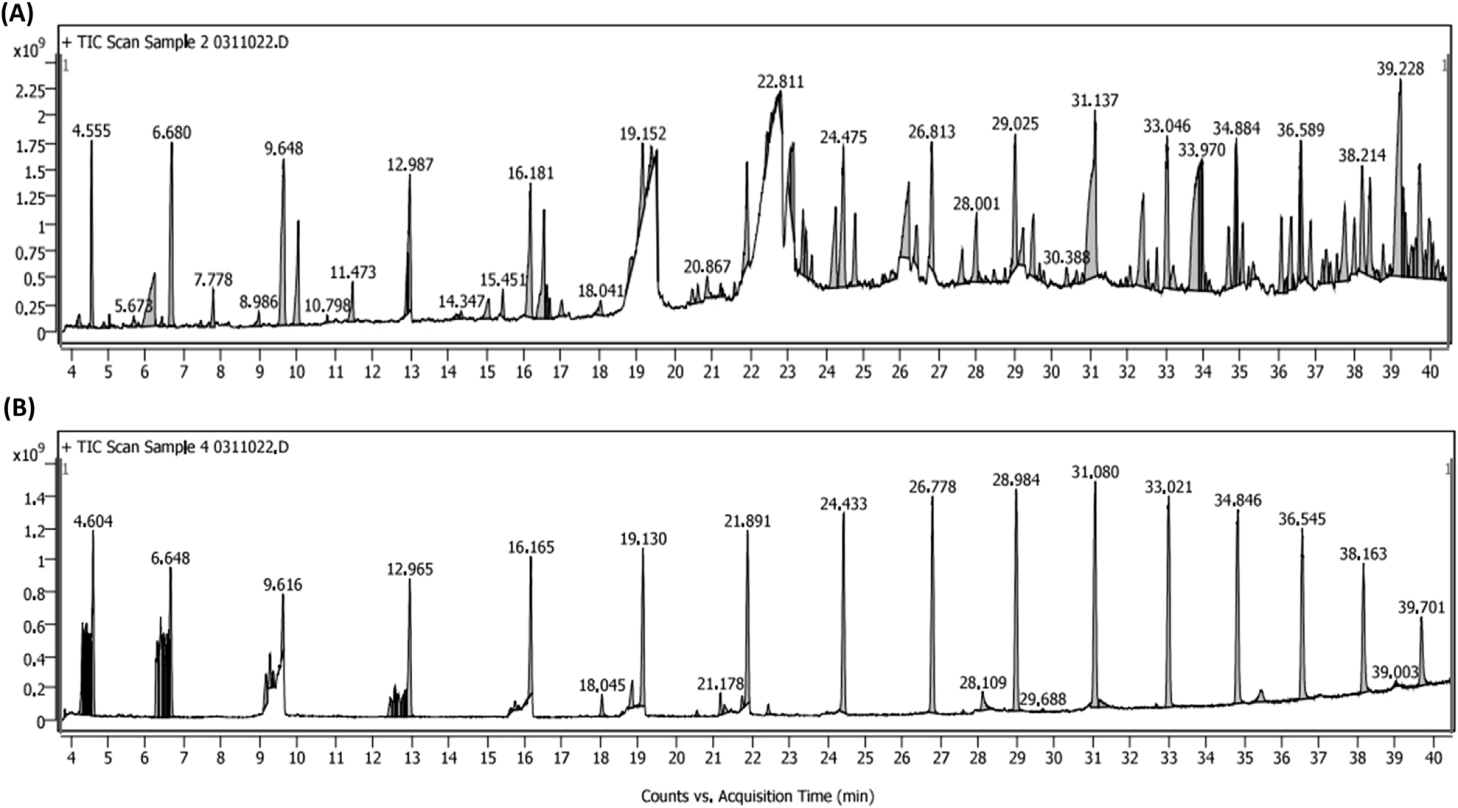
**(A)** GC-MS chromatogram of *Alcea rose*a ethyl acetate extract. **(B)** GC-MS chromatogram of *Alcea rosea* methanol extract.

Chromatograms of metabolites identified in the Alcea rosea ethyl acetate and methanol extracts are shown in (Figure 4). Some of the major metabolites identified in the aforementioned extracts are Nonanoic Acid, N-Hexadecenoic Acid, Eicosanoic Acid, Octadecanoic Acid, Propionic Acid, Hentriacontane, Kaempferol 3-O-(6″-Galloyl)-Beta-D-Glucopyranoside, Phytol, α-Tocopherol, 1-Beta-D-Ribofuranosyl-3-[5-Tetraazolyl]-1,2,4-Triazole, Ergosterol and Stigmasterol etc. (Supplementary Table 1). While the present study employed GC-MS for qualitative metabolite profiling, quantitative analysis of key compounds such as kaempferol and phytol using HPLC-DAD/LC-MS, along with batch-to-batch reproducibility and bioactivity correlation, are part of our ongoing and future research.

### 3.2 *In vivo* studies

#### 3.2.1 AR-EA and AR-Met inhibit LPS induced inflammatory response (Paw histopathology)

**FIGURE 5:**
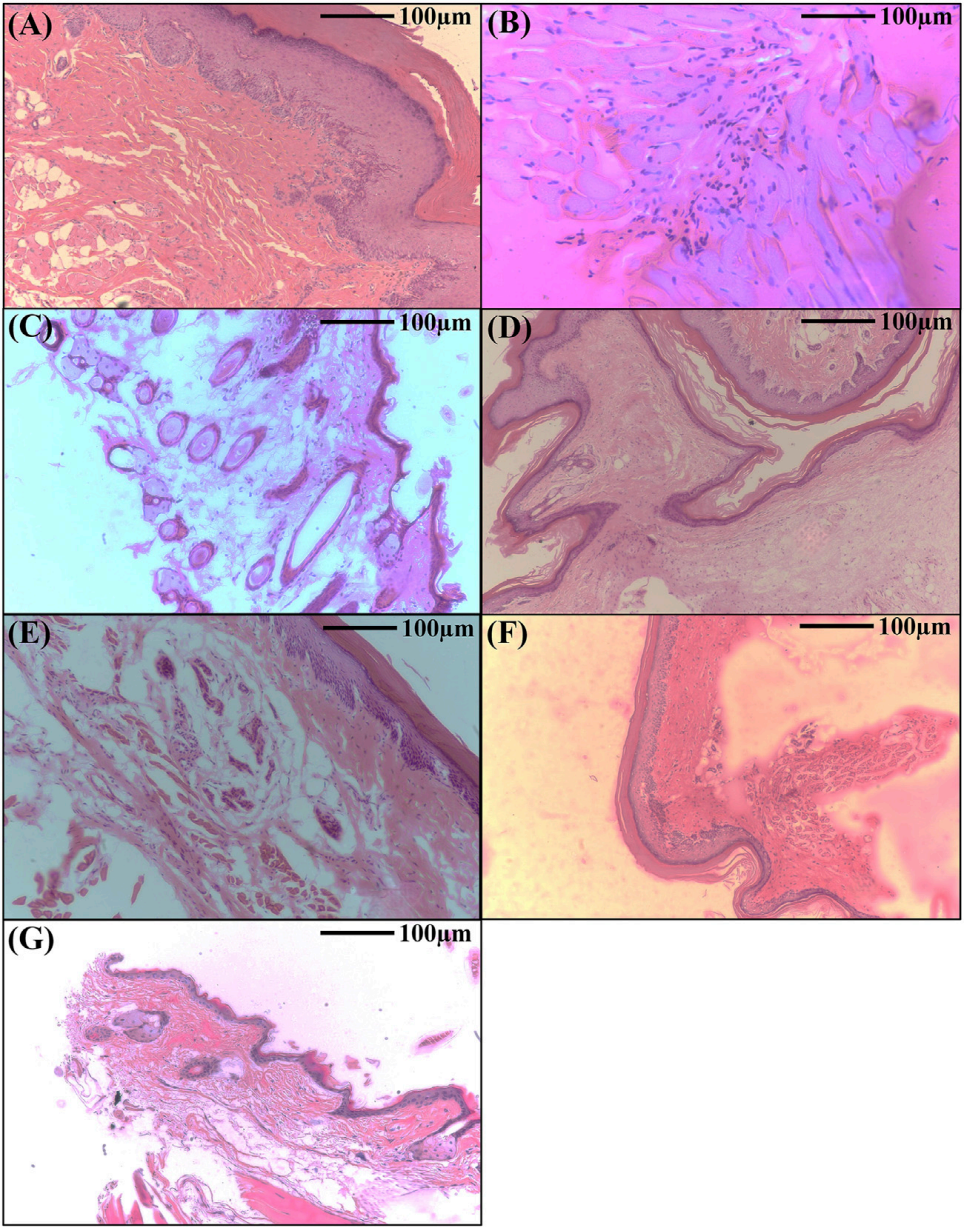
Histopathological analysis showing the therapeutic effects of *Alcea rosea* during LPS induced paw edema in mice. **(A)** Normal control; **(B)** LPS treated; **(C)** LPS + AR-EA treated (25 mg/kgbwt); **(D)** LPS + AR-EA treated (50 mg/kgbwt); **(E)** LPS + AR-Met treated (25 mg/kgbwt); **(F)** LPS + AR-Met treated (50 mg/kgbwt); **(G)** LPS + dexamethasone treated.

Administering lipopolysaccharide (LPS) into the sub-plantar area of the right hind paw in mice triggers a rapid inflammatory reaction. The histopathological analysis of the control group showed intact dermis and epidermis with no signs of lesion development, cellular infiltration, or edema. Nevertheless, the group that received only LPS had significant vasodilation and swelling, accompanied with neutrophil migration into the gaps between cells, collagen fibers, and blood vessels (Figure 5). On the other hand, mice who received ethyl acetate extract and methanolic extract at dosages of 25 and 50 mg/kg body weight exhibited a decrease in swelling and a decrease in the presence of polymorphonuclear (PMN) cells infiltrating the affected area. This reduction occurred in a way that was dependent on the dosage administered. Furthermore, these groups had reduced vascular congestion and epidermal thickening. The anti-inflammatory effects of the highest dosages of AR-EA and AR-Met were almost like those noted in the Dexamethasone-treated group, which was used as a positive control (Figure 5). While representative histological images were included to illustrate tissue-level changes, quantitative histopathological scoring was not performed, as the study prioritized molecular and biochemical assessments to evaluate inflammation.

#### 3.2.2 Effect of AR-EA and AR-Met on haematological parameters of mice models

This study investigated the impact of LPS-induced inflammation on a comprehensive panel of blood markers. We measured total and direct bilirubin, liver enzyme activities (AST and ALT), alkaline phosphatase, total protein profile (including albumin and globulin), kidney function markers (blood urea, blood urea nitrogen, and creatinine), uric acid, serum appearance, cholesterol, triglycerides, and C-reactive protein across all groups. These markers are known to be sensitive to changes caused by inflammation. Compared to the normal control group (receiving only saline), animals in the LPS group displayed a significant increase (*p* < 0.001) in all measured parameters except serum uric acid, which decreased. Conversely, animals in the AR-EA and AR-Met groups showed a significant improvement (*p* < 0.001) as evidenced by a decrease in all measured parameters and an increase in serum uric acid, as detailed in (Table 2).

**TABLE 2:** Effects of *Alcea rosea* extracts on liver function, kidney function, lipid profile, and inflammation marker in LPS-induced inflammation model.

Tests	Normal group	LPS only		LPS + Dexa	LPS + Ar-EA		LPS + AR-Met	
					25 mg/kg bwt	50 mg/kg bwt	25 mg/kg bwt	50 mg/kg bwt
Total bilirubin (mg/dL)	0.01 ± 0.0003	1.01 ± 0.0374****		0.11 ± 0.0051***	0.16 ± 0.0069**	0.13 ± 0.0033****	0.17 ± 0.0192*	0.15 ± 0.0061***
Bilirubin (Direct) mg/dL	0.04 ± 0.0019	2.97 ± 0.0874***		0.06 ± 0.0023****	0.10 ± 0.0044***	0.08 ± 0.0021****	0.13 ± 0.0081*	0.09 ± 0.0026***
AST (IU/L)	21.0 ± 0.69	213.0 ± 8.82***		41.0 ± 1.56****	60.0 ± 2.88****	51.0 ± 1.30***	66.0 ± 3.91*	54.0 ± 2.14**
ALT (IU/L)	32.0 ± 0.77	134.0 ± 3.97****		39.2 ± 1.55****	49.0 ± 4.01*	43.4 ± 2.30**	46.8 ± 4.78*	54.0 ± 2.61***
Alkaline phosphatase (IU/L)	124.0 ± 5.33	284.0 ± 11.60***		138.0 ± 4.82***	172.0 ± 6.60**	164.0 ± 8.73***	159.0 ± 6.85**	187.0 ± 8.26*
Total proteins (gms/dL)	8.0 ± 0.18	15.5 ± 0.77****		9.6 ± 0.35****	11.25 ± 0.42***	10.17 ± 0.90**	11.65 ± 1.53*	10.34 ± 0.34***
Serum albumin (gms/dL)	3.8 ± 0.12	11.3 ± 0.43****		4.1 ± 0.15****	4.91 ± 0.38*	4.65 ± 0.81***	5.8 ± 1.42^NS^	4.94 ± 0.21***
Serum globulin (gms/dL)	2.1 ± 0.06	4.4 ± 0.15****		2.9 ± 0.10****	3.71 ± 0.15**	3.44 ± 0.12**	3.80 ± 0.14****	3.59 ± 0.11****
A/G ratio	1.8 ± 0.06	2.5 ± 0.08		1.4 ± 0.05	1.3 ± 0.04	1.4 ± 0.07	1.5 ± 0.07	1.3 ± 0.05
Mean values of mice groups								
Blood urea (mgs/dL)	28.9 ± 0.92	77.6 ± 3.10***		32.6 ± 1.30***	40.2 ± 2.81**	33.4 ± 1.70***	48.2 ± 3.41*	34.3 ± 1.29***
Blood urea nitrogen (mg/dL)	13.4 ± 0.44	32.2 ± 1.29****		16.0 ± 0.64***	17.6 ± 1.84**	16.8 ± 0.97****	19.8 ± 1.49**	17.2 ± 0.60***
Serum creatinine (mg/dL)	0.09 ± 0.003	0.25 ± 0.011****		0.13 ± 0.005****	0.19 ± 0.008***	0.16 ± 0.019*	0.21 ± 0.009***	0.18 ± 0.007***
Serum uric acid (mgs/dL)	2.2 ± 0.08	0.18 ± 0.01****		1.9 ± 0.07****	1.28 ± 0.05***	1.84 ± 0.06****	0.98 ± 0.04***	1.3 ± 0.05**
Lipid profile of all mice groups: Cholesterol/Triacylglycerol-TAG-Serum								
Serum appearance		Clear	Not clear	Clear	Clear	Clear	Clear	Clear
Serum cholesterol (Total) mg/dL		101.9 ± 3.57	143.3 ± 5.29***	104.3 ± 5.64*	112.1 ± 4.10***	102.3 ± 6.06***	119.8 ± 5.67**	114.7 ± 4.24***
Serum triglycerides (mg/dL)		90.0 ± 2.77	142.5 ± 5.05***	117.6 ± 4.11****	126.4 ± 4.89**	119.1 ± 4.06**	133.2 ± 7.06^NS^	128.6 ± 4.47**
C-reactive protein of all mice groups: C-reactive protein (Quantitative)								
C-reactive protein (mg/L)	0.02 ± 0.001	0.12 ± 0.005***	0.03 ± 0.001***	0.06 ± 0.008*	0.04 ± 0.007****	0.09 ± 0.004**		0.07 ± 0.003***

NS, not significant; AR-Ea, *Alcea rosea* Ethyl acetate extract; AR-Met, *Alcea rosea* methanol extract; LPS, lipopolysaccharide; AST, aspartate transferase; ALT, alanine transaminase; KFT, kidney function test; A/G ratio, Albumin/Globulin ratio. Data is presented as mean ± SEM (*n* = 6).

*p < 0.05, **p < 0.01, **p < 0.001, p**** <0.0001 vs. Normal group.

## 4 Discussion

The study aimed to evaluate the anti-inflammatory and anti-colorectal cancer properties of *Alcea rosea* extracts. Various parameters including protein denaturation, nitric oxide production, HRBC membrane stabilization, and gene expression were investigated. Despite its traditional medicinal use, scientific validation is lacking. The protein denaturation assay revealed ethyl acetate and methanol extracts as potent inhibitors. Similar results were observed in many earlier studies evaluating the protein denaturation-inhibiting property of medicinal herbs during inflammation, such as *Barringtonia racemosa*, *Cedrus libani*, and *Pinus brutia* (Osman et al., 2016). Our results indicate that the ethyl acetate (AR-EA) and (AR-Met) methanolic extracts of *Alcea rosea* might be potent inhibitors of protein denaturation. Major metabolites identified in the *Alcea rosea* extracts are Nonanoic Acid, N-Hexadecenoic Acid, Eicosanoic Acid, Octadecanoic Acid, Propionic Acid, Hentriacontane, Kaempferol 3-O-(6″-Galloyl)-Beta-D-Glucopyranoside, Phytol, α-Tocopherol, 1-Beta-D-Ribofuranosyl-3-[5-Tetraazolyl]-1,2,4-Triazole, Ergosterol, and Stigmasterol (Supplementary Table 1). Kaempferol 3-O-(6″-galloyl)-β-D-glucopyranoside identified in this study was previously isolated and structure-activity validated in our published work (Parry et al., 2025). These metabolites have demonstrated anti-inflammatory properties by downregulating pro-inflammatory cytokines and signalling pathways and exhibit promising anticancer activities by targeting multiple hallmarks of cancer including apoptosis induction, cell cycle arrest, and inhibition of metastasis (Zhang et al., 2022; Lian et al., 2023; Es-Sai et al., 2025; Parry et al., 2025). Among the identified metabolites, kaempferol 3-O-(6″-galloyl)-β-D-glucopyranoside has been reported to exert potent anti-inflammatory effects by inhibiting NFκB signalling. Similarly, α-Tocopherol is known for its antioxidant and anti-inflammatory actions (Sharifi-Rad et al., 2022a; Benmohamed et al., 2023), and phytol has been implicated in modulating inflammatory cytokines (Sharifi-Rad et al., 2022b). These findings provide a mechanistic basis for the observed biological effects of the extracts.

Under inflammatory conditions, the uncontrolled activity of proteinases can unleash havoc on surrounding tissues, leading to their destruction. This phenomenon is readily observed in rheumatoid arthritis, where matrix metalloproteinase’s (MMPs) act as relentless demolition crews, dismantling the integrity of joint (Karrat et al., 2022). Similarly, in Crohn’s disease and ulcerative colitis, MMPs accumulate on the intestinal lining, fuelling chronic inflammation (Page-McCaw et al., 2007). Cancer cells, in their relentless quest for growth and survival, hijack the power of proteinases for their nefarious purposes. By amplifying the expression of these enzymes, cancerous cells acquire the ability to invade surrounding tissues, establish a network of blood vessels for nourishment (angiogenesis), and even evade the watchful sentry of the immune system (O’Sullivan et al., 2015). Various proteinases contribute to tumour progression, (Cuffaro et al., 2024), with cathepsins aiding antigen presentation and causing tissue damage, (López-Otín and Matrisian, 2007), caspases facilitating programmed cell death (Chang et al., 2023), ADAMs influencing cell signalling and metastasis, and uPA orchestrating extracellular matrix degradation for invasion (Yaowachai et al., 2023). Researchers aim to develop strategies to counteract these proteinases. Testing *Alcea rosea* extracts, ethyl acetate showed the highest antiproteinase activity, possibly due to protease inhibitors. Similar findings were observed in studies on *Rhinacanthus nasutus* and *Tamilnadia uliginosa* extracts. Understanding proteinase intricacies drives innovative therapies for inflammatory and cancerous conditions. Similar membrane-stabilizing effects have been reported for *Hibiscus sabdariffa*, another Malvaceae family member, further supporting the ethnopharmacological relevance of *Alcea rosea* (Ali et al., 2005).

Inflammation involves leukocyte migration and lysosomal enzyme release, causing cellular damage (Kommu et al., 2023). Synthetic drugs or plant extracts preventing red blood cell haemolysis indicate anti-inflammatory and anticancer potential (Joseph et al., 2013). These agents stabilize membranes, inhibit enzymes, and act as antioxidants (Haidari et al., 2023). Understanding their mechanisms and exploring synergies could lead to innovative therapies. *Alcea rosea* extracts, especially ethyl acetate and methanolic, showed potent membrane stabilization, suggesting they could inhibit leukocyte membrane lysis, reducing inflammation. Notably, a comparable observation was noted by Belkhodja (Belkhodja et al., 2024) in their study exploring the anti-inflammatory efficacy of *Malva sylvestris* extract, further confirming the potential of plant-based extracts for mitigating inflammatory responses. Inflammatory reactions include the translocation of leukocytes from the circulatory system to the impacted region via extravasation. These immune cells secrete lysosomal enzymes, such as bactericidal proteins and proteases, which induce cellular damages by deteriorating membranes. The breakdown of this barrier heightens vulnerability to oxidative stress and lipid peroxidation. Given that red blood cell (RBC) membranes have structural resemblances to lysosomal membranes, the capacity of synthetic medicines or botanical extracts to inhibit RBC hemolysis is a vital indication of their anti-inflammatory efficacy. The ethyl acetate and methanolic extracts of *Alcea rosea* exhibited the greatest efficacy in inhibiting RBC membrane hemolysis, indicating their function in membrane stability. These extracts may also impede leukocyte membrane lysis, the release of phospholipase A2 (PLA2) enzymes, and resulting in synthesis of inflammatory mediators. Wasti and Coworkers obtained similar results when evaluating the anti-inflammatory properties of *Ajugarin* I and *Ajuga bracteosa* extracts (Wasti et al., 2024).

The anti-inflammatory results of AR-EA and AR-Met were verified in BALB/c mice model. Where, AR-EA and AR-Met significantly ameliorated the LPS induced inflammation in Mice paw tissue. Similar results were observed by Sawhney (Sawhney et al., 2022) while checking the effect of Arteannuin-B and (3-Chlorophenyl)-2-Spiroisoxazoline Derivative in LPS induced BALB/c mice.

Over-expression of iNOS can cause excess NO production, harming cells and inducing pro-inflammatory factors. NO reacts with free radicals, forming peroxy-nitrite, further damaging cells (Huang et al., 2023). Our research provides compelling evidence that ethyl acetate and methanolic extracts of *Alcea rosea* exhibit a concentration-dependent decrease in NO levels. This potential inhibitory effect suggests the presence of yet-to-be-identified nitric oxide inhibitors within this novel medicinal plant. Similar results were achieved by Ginovyan and co-workers who investigated the effect of extracts from *Rumex obtusifolius* on nitric oxide inhibition (Ginovyan et al., 2023).

COX-2 catalyses prostaglandin synthesis from arachidonic acid, contributing to inflammation’s hallmark features (Bogdan, 2001). We observed a significant decrease in the protein levels of COX-2 in both HCT116 and HT29 cells following treatment with AR-EA, and AR-Met extracted from *A. rosea*. Previously, various researchers have shown that many plant extracts exert anti-inflammatory (Wan et al., 2024) and anti-colorectal cancer (Siddiqui, 2011) effects by decreasing COX-2 protein levels.

NF-κB mediates inflammation and colorectal cancer progression, regulating genes for inflammation, cell proliferation, and apoptosis evasion, promoting tumour growth (Lin et al., 2022). We observed a significant decrease in the protein levels of NFκB in both HCT116 and HT29 cells following treatment with AR-EA, and AR-Met extracted from *A. rosea*.

PPAR-γ has anti-inflammatory effects by inhibiting NF-κB and regulating cytokines. In CRC, it acts as a tumour suppressor, regulating cell processes (Hayden and Ghosh, 2008). Many plant extracts have been shown to have anti-inflammatory (Mueller et al., 1998) and anti-colorectal cancer (Rios-Avila et al., 2012) effects by decreasing PPAR-γ protein levels.

## 5 Conclusion

*Alcea rosea* possesses significant anti-inflammatory and anti-colorectal cancer potential. The lack of documented toxicity associated with *Alcea rosea* despite its long-standing use in traditional medicine supports its safety. Among the five extracts of *Alcea rosea*, AR-EA and AR-Met exhibited the most effective responses in terms of protein denaturation, nitric oxide surge, HRBC haemolysis, proteinase inhibition, and CRC cell viability, as depicted in the results section. Moreover, AR-EA and AR-Met significantly decreased the protein levels of key inflammatory genes, such as COX-2, NFκB, and PPARγ in CRC cells and reduced LPS-induced paw inflammation in BALB/c mice, which are known to play pivotal roles in various inflammatory pathways and tumorigenicity. Consistent with our MTT results, which revealed that the AR-EA and AR-Met extracts have significant anti-proliferative effects, our DAPI-PI and Western blotting results for C-PARP and cleaved Caspase 3 confirmed that these extracts can promote apoptosis in a significant proportion of HCT116 and HT29 colorectal cancer cells. Future studies will utilize pathway-specific inhibitors, NFκB translocation assays, cytokine profiling, and CRC-relevant murine models (such as AOM/DSS-induced CRC) to validate the mechanistic dependencies and therapeutic relevance of the isolated metabolites. Additionally, immunohistochemistry for inflammatory markers like COX-2 and TNF-α is planned to further support the histological observations. Given the traditional use of *Alcea rosea* and absence of toxicity, the identified metabolites warrant further development as potential therapeutic leads. The therapeutic potential of these extracts may lie in their diverse modes of action. They can operate independently or synergistically at various molecular targets within intricate signalling networks, ultimately dampening the effector pathways associated with inflammation related colorectal cancer.

## Data Availability

The original contributions presented in the study are included in the article/Supplementary Material, further inquiries can be directed to the corresponding authors.

## Abbreviations

AR, Alcea rosea; EA, Ethyl acetate; Met, Methanol; MTT, 3-(4,5-Dimethylthiazol-2-yl)-2,5-Diphenyltetrazolium Bromide; HCT116, Human colorectal carcinoma cell line; HT29, Human colorectal adenocarcinoma cell line; DAPI, 4′,6-diamidino-2-phenylindole; CRC, Colorectal cancer; COX-2, Cyclooxygenase-2; NFκB, Nuclear factor-κB; PPAR-γ, Peroxisome proliferator-activated receptor gamma; PARP, Poly (ADP-ribose) polymerase.
