# Supplementary material for: Anti-inflammatory and anticancer properties of Alcea rosea extracts: Insights from in vitro and in vivo studies
Source: Front Pharmacol. 2025 Jul 9;16:1595604. doi: 10.3389/fphar.2025.1595604 (PMC12283617; doi:10.3389/fphar.2025.1595604)
Supplement: Supplementary file 2 [file Supplementaryfile1.docx]

**Supplementary Table 1:** Compounds identified in *Alcea rosea* ethyl acetate extract (AR-EA) (GC-MS study).

| **S.no** | **Name** | **Formula** | **RT** | **Mass** | **CAS** | **%Area** |
| --- | --- | --- | --- | --- | --- | --- |
|  | Decanoic acid, 3-hydroxy, methyl ester | C_11_H_22_O_3_ | 4.232 | 202.2906 | 56618-58-7 | 3.59 |
|  | Propionic Acid | C_3_H_6_O_2_ | 4.555 | 74.08 | 79-09-4 | 6.17 |
|  | Decanoic acid, silver (1+) salt | C_10_H_19_AgO_2_ | 4.882 | 279.12 | 13126-67-5 | 1.42 |
|  | 2-Azido-2,4,4,6,6-pentamethylheptane | C_12_H_25_N_3_ | 5.027 | 211.35 | 1000293-29-0 | 1.36 |
|  | Silane dimethyl [(methylsilyl)methyl]- | C_4_H_14_Si_2_ | 5.673 | 181.3250 | 18148-13-5 | 2.84 |
|  | Nonanoic acid | C_9_H_18_O_2_ | 6.232 | 158.23 | 112-05-0 | 34.16 |
|  | Methyl 12,13 tetradecadienoate | C_15_H_26_O_2_ | 6.425 | 238.37 | 1000336-337 | 1.66 |
|  | Cyclohexasiloxane, dodecmethyl | C_10_H_36_O_6_S_i6_ | 6.680 | 444.92 | 540-97-6 | 47.06 |
|  | Decanoic acid, silver (1+) salt | C_10_H_19_AgO_2_ | 7.452 | 279.12 | 13126-67-5 | 1.41 |
|  | Trans-2-methyl-4-n-pentylthiane, S, S-dioxide | C_11_H_22_O_2_S | 7.778 | 218.36 | 1000215-75-3 | 6.85 |
|  | Octadecane, 1-isocyanato- | C_19_H_37_NO | 8.986 | 295.5 | 112-96-9 | 3.56 |
|  | Cycloheptasiloxane, tetradecamethyl | C_14_H_42_O_7_Si_7_ | 9.648 | 519.0776 | 107-50-6 | 55.62 |
|  | 4-Diethylaminophenyl isothiocyanate | C_11_H_14_N_2_S | 10.032 | 178.26 | 84381-54-4 | 25.94 |
|  | Cis-2-Methyl-4-n-pentylthiane, S, S-dioxide | C_11_H_22_O_2_S | 10.798 | 218.36 | 1000215-67-9 | 1.04 |
|  | Trans-2-methyl-4-n-pentylthiane, S, S-dioxide | C_11_H_22_O_2_S | 11.473 | 218.36 | 1000215-75-3 | 9.55 |
|  | Cyclooctasiloxane, hexadecamethyl | C_16_H_48_O_8_Si_8_ | 12.926 | 593.2 | 556-68-3 | 8.65 |
|  | Cyclooctasiloxane, hexadecamethyl | C_16_H_48_O_8_Si_8_ | 12.987 | 593.2 | 556-68-3 | 31.86 |
|  | 4,5,5,7-Tetrahydroxydecyl isothiocyanate | C_11_H_21_NO_4_S | 14.215 | 263.119 | 57103-43-2 | 1.56 |
|  | Trans-2-methyl-4-n-pentylthiane, S, S-dioxide | C_11_H_22_O_2_S | 14.347 | 218.36 | 100021575-3 | 1.64 |
|  | Tetradecanoic acid | C_14_H_28_O_2_ | 15.077 | 228.37 | 544-63-8 | 7.44 |
|  | Trans-2-methyl-4-n-pentylthiane, S, S-dioxide | C_11_H_22_O_2_S | 15.451 | 218.36 | 1000215-75-3 | 5.02 |
|  | Cyclononasiloxane, octadecamethyl | C_18_H_54_O_9_Si_9_ | 16.181 | 667.4 | 556-71-8 | 40.14 |
|  | 2-Pentadecanone,6,10,14-trimethyl | C_18_H_36_O | 16.543 | 268.477 | 502-69-2 | 33.29 |
|  | Becteriochlporophyll-c-stearyl | C_52_H_72_MgN_4_O_4_ | 16.611 | 841.5 | 1000164-49-7 | 6.75 |
|  | Becteriochlporophyll-c-stearyl | C_52_H_72_MgN_4_O_4_ | 16.688 | 841.5 | 1000164-49-7 | 3.67 |
|  | Cyclopentyl-methyl-phosphinic acid,2-isopropyl-5-methyl-cyclohexyl ester | C_16_H_31_O_2_P | 17.011 | 286.39 | 1000194-56-2 | 4.32 |
|  | 5-(2-Oxo- [1,2] oxathiolane-3-yl) pentanoic acid, methyl ester | C_19_H_16_O_4_S | 18.041 | 340.4 | 1000186-41-5 | 5.17 |
|  | n-Hexadecanoic acid | C_16_H_32_O_2_ | 18.871 | 256.42 | 57-10-3 | 5.83 |
|  | Unknown |  | 19.152 |  |  | 22.46 |
|  | n-Hexadecanoic acid | C_16_H_32_O_2_ | 19.395 | 256.42 | 57-10-3 | 7.84 |
|  | n-Hexadecanoic acid | C_16_H_32_O_2_ | 19.715 | 256.42 | 57-10-3 | 8.13 |
|  | Nona-2,3-dienoic acid, ethyl ester | C_11_H_18_O_2_ | 20.467 | 182.26 | 1000187-19-2 | 4.03 |
|  | Bacteriochlorophyll-c-stearyl | C_52_H_72_MgN_4_O_4_ | 20.615 | 841.5 | 1000164-49-7 | 3.84 |
|  | Bacteriochlorophyll-c-stearyl | C_52_H_72_MgN_4_O_4_ | 20.867 | 841.5 | 1000164-49-7 | 6.55 |
|  | Methyl 9,10-octadecadienoate | C_19_H_34_O_2_ | 21.219 | 294.5 | 100033645-7 | 2.39 |
|  | Eicosanoic Acid | C_20_H_40_O_2_ | 21.317 | 312.53 | 506-30-9 | 1.01 |
|  | Bacteriochlorophyll-c-stearyl | C_52_H_72_MgN_4_O_4_ | 21.594 | 841.5 | 1000164-49-7 | 2.18 |
|  | Kaempferol 3-O-(6''-galloyl)-beta-D-glucopyranoside | C_28_H_24_O_15_ | 21.917 | 600.5 | [56317-05-6](https://www.chemsrc.com/en/baike/305502.html) | 27.54 |
|  | Methyl 8,9-octadecadienoate | C_19_H_34_O_2_ | 22.456 | 294.5 | 1000336-40-0 | 5.92 |
|  | Methyl 8,9-octadecadienoate | C_19_H_34_O_2_ | 22.572 | 294.5 | 1000336-40-0 | 2.88 |
|  | Methyl 7,8-octadecadienoate | C_19_H_34_O_2_ | 22.811 | 294.5 | 1000336-44-6 | 6.09 |
|  | Octadecanoic acid | C_18_H_36_O_2_ | 23.067 | 284.5 | 57-11-4 | 5.49 |
|  | Octadecanoic acid | C_18_H_36_O_2_ | 23.157 | 284.5 | 57-11-4 | 24.42 |
|  | 1-1`-Biphenyl, 5-hydroxy-3,2`,3`,4`-tetramethoxy- | C_16_H_18_O_5_ | 23.406 | 290.31 | 119101-28-9 | 14.28 |
|  | 2-Butanone,4-(2,2,6-trimethylcyclohexyl)- | C_13_H_24_O | 23.483 | 196.33 | 6138-85-8 | 8.81 |
|  | Nona-2,3-dienoic acid, ethyl ester | C_11_H_18_O_2_ | 23.638 | 182.26 | 1000187-19-2 | 4.20 |
|  | 1,2-Oxathiane,6-dodecyl-,2,2-dioxide | C_16_H_32_O_3_S | 24.268 | 304.5 | 15224-88-1 | 29.74 |
|  | Kaempferol 3-O-(6''-galloyl)-beta-D-glucopyranoside | C_28_H_24_O_15_ | 24.475 | 600.5 | [56317-05-6](https://www.chemsrc.com/en/baike/305502.html) | 46.74 |
|  | Trihexadecyl borate | C_48_H_99_BO_3_ | 24.785 | 735.1 | 2665-11-4 | 18.74 |
|  | 2,6,6-Trimethyl-9-undecen-1-ol | C_14_H_28_O | 25.246 | 212.37 | 1000131-31-6 | 1.38 |
|  | beta-d-Mannofuranose,2,3:5,6-di-O-ethylboranediyl-1-O-(10-undecen-1-yl)- | C_21_H_38_B_2_O_6_ | 25.515 | 408.1 | 1000155-22-1 | 1.39 |
|  | beta-d-Mannofuranose,2,3:5,6-di-O-ethylboranediyl-1-O-(10-undecen-1-yl)- | C_21_H_38_B_2_O_6_ | 25.576 | 408.1 | 1000155-22-1 | 1.44 |
|  | beta-d-Mannofuranose,2,3:5,6-di-O-ethylboranediyl-1-O-(10-undecen-1-yl)- | C_21_H_38_B_2_O_6_ | 25.754 | 408.1 | 1000155-22-11 | 3.77 |
|  | Eicosanoic acid | C_20_H_40_O_2_ | 26.202 | 312.5 | 506-30-9 | 35.44 |
|  | Trihexadecyl borate | C_48_H_99_BO_3_ | 26.419 | 735.1 | 2665-11-4 | 10.86 |
|  | Bacteriochlorophyll-c-stearyl | C_25_H_72_MgM_4_O_4_ | 27.618 | 841.5 | 1000164-49-7 | 9.12 |
|  | Bacteriochlorophyll-c-stearyl | C_25_H_72_MgM_4_O_4_ | 27.627 | 841.5 | 1000164-49-7 | 11.14 |
|  | Trihexadecyl borate | C_48_H_99_BO_3_ | 28.001 | 735.1 | 2665-11-4 | 20.48 |
|  | Bacteriochlorophyll-c-stearyl | C_25_H_72_MgM_4_O_4_ | 28.069 | 841.5 | 1000164-49-7 | 2.29 |
|  | Bacteriochlorophyll-c-stearyl | C_25_H_72_MgM_4_O_4_ | 28.247 | 841.5 | 1000164-49-7 | 1.41 |
|  | Bacteriochlorophyll-c-stearyl | C_25_H_72_MgM_4_O_4_ | 28.751 | 841.5 | 1000164-49-7 | 2.56 |
|  | Docosanoic acid | C_22_H_44_O_2_ | 29.142 | 340.6 | 112-85-6 | 8.34 |
|  | Trihexadecyl borate | C_48_H_99_BO_3_ | 39.510 | 735.1 | 2665-11-4 | 17.59 |
|  | beta-d-Mannofuranose,2,3:5,6-di-O-ethylboranediyl-1-O-(10-undecen-1-yl)- | C_21_H_38_B_2_O_6_ | 29.671 | 408.1 | 1000155-22-1 | 2.78 |
|  | Bacteriochlorophyll-c-stearyl | C_25_H_72_MgM_4_O_4_ | 29.771 | 841.5 | 1000164-49-7 | 2.53 |
|  | E-8-Methyl-9-tetradecen-1-ol acetate | C_17_H_32_O_2_ | 30.388 | 268.4 | 130814 | 4.98 |
|  | beta-d-Mannofuranose,2,3:5,6-di-O-ethylboranediyl-1-O-(10-undecen-1-yl)- | C_21_H_38_B_2_O_6_ | 30.646 | 408.1 | 1000155-22-1 | 3.27 |
|  | beta-d-Mannofuranose,2,3:5,6-di-O-ethylboranediyl-1-O-(10-undecen-1-yl)- | C_21_H_38_B_2_O_6_ | 30.798 | 408.1 | 1000155-22-1 | 2.22 |
|  | Trihexadecyl borate | C_48_H_99_BO_3_ | 31.137 | 735.1 |  | 99.54 |
|  | beta-d-Mannofuranose,2,3:5,6-di-O-ethylboranediyl-1-O-(10-undecen-1-yl)- | C_21_H_38_B_2_O_6_ | 31.816 | 408.1 | 1000155-22-1 | 1.05 |
|  | beta-d-Mannofuranose,2,3:5,6-di-O-ethylboranediyl-1-O-(10-undecen-1-yl)- | C_21_H_38_B_2_O_6_ | 31.958 | 408.1 | 1000155-22-1 | 1.69 |
|  | Palmitoyl chloride | C_16_H_31_ClO | 32.061 |  | 112-67-4 | 4.48 |
|  | Trihexadecyl borate | C_48_H_99_BO_3_ | 32.423 | 735.1 | 2665-11-4 | 37.95 |
|  | Bacteriochlorophyll-c-stearyl | C_25_H_72_MgM_4_O_4_ | 32.539 | 841.5 | 1000164-49-7 | 7.78 |
|  | beta-d-Mannofuranose,2,3:5,6-di-etylboranediyl-cis-nerolidyl | C_25_H_42_B_2_O_6_ | 32.781 | 460.2 | 1000155-68-3 | 7.92 |
|  | Kaempferol 3-O-(6''-galloyl)-beta-D-glucopyranoside | C_28_H_24_O_15_ | 33.046 | 600.5 | [56317-05-6](https://www.chemsrc.com/en/baike/305502.html) | 45.95 |
|  | beta-d-Mannofuranose,2,3:5,6-di-O-ethylboranediyl-1-O-(10-undecen-1-yl)- | C_21_H_38_B_2_O_6_ | 32.211 | 408.1 | 1000155-22-1 | 8.87 |
|  | beta-d-Mannofuranose,2,3:5,6-di-O-ethylboranediyl-1-O-(10-undecen-1-yl)- | C_21_H_38_B_2_O_6_ | 33.430 | 408.1 | 1000155-22-1 | 1.14 |
|  | Trihexadecyl borate | C_48_H_99_BO_3_ | 33.892 | 735.1 | 2665-11-4 | 80.17 |
|  | Hentriacontane | C_31_H_64_ | 33.950 | 436.8 | 630-04-6 | 29.10 |
|  | Trihexadecyl borate | C_48_H_99_BO_3_ | 33.970 | 735.1 | 2665-11-4 | 17.47 |
|  | Phytol | C_20_H_40_O | 34.080 | 296.5 | 150-86-7 | 2.66 |
|  | Fumaric acid, decyl propargyl ester | C_17_H_26_O_4_ | 34.173 | 294.4 | 1000330-53-4 | 2.62 |
|  | Didodecylphosphine oxide | C_24_H_51_O_3_P^+^ | 34.671 | 417.6 | 21302-09-0 | 17.48 |
|  | Tetracosamethyl-cyclododecasiloxane | C_24_H_72_O_12_Si_12_ | 34.871 | 889.8 | 18919-94-3 | 23.72 |
|  | Tetracosamethyl-cyclododecasiloxane | C_24_H_72_O_12_Si_12_ | 34.884 | 889.8 | 18919-94-3 | 21.24 |
|  | Trihexadecyl borate | C_48_H_99_BO_3_ | 35.048 | 735.1 | 2665-11-4 | 12.55 |
|  | Bacteriochlorophyll-c-stearyl | C_25_H_72_MgM_4_O_4_ | 35.207 | 841.5 | 1000164-49-7 | 2.55 |
|  | Unknown |  | 35.326 |  |  | 4.11 |
|  | 7-Isopropyl-10-methyl-1-oxo-1,5-dithia-spiro [5.5] undecane-2-carboxylic acid | C_14_H_24_O_3_S_2_ | 36.079 | 304.5 | 1000189-33-1 | 18.16 |
|  | beta-d-Mannofuranose,2,3:5,6-di-etylboranediyl-cis-nerolidyl | C_25_H_42_B_2_O_6_ | 36.205 | 460.2 | 1000155-68-3 | 3.44 |
|  | Trihexadecyl borate | C_48_H_99_BO_3_ | 36.340 | 735.1 | 2665-11-4 | 22.04 |
|  | Bacteriochlorophyll-c-stearyl | C_25_H_72_MgM_4_O_4_ | 36.389 | 841.5 | 1000164-49-7 | 1.94 |
|  | Tetracosamethyl-cyclododecasiloxane | C_24_H_72_O_12_Si_12_ | 36.570 | 889.8 | 18919-94-3 | 18.86 |
|  | Tetracosamethyl-cyclododecasiloxane | C_24_H_72_O_12_Si_12_ | 36.589 | 889.8 | 18919-94-3 | 24.18 |
|  | Vitamin E | C_29_H_50_O_2_ | 36.851 | 430.7 | 59-02-9 | 15.72 |
|  | Bacteriochlorophyll-c-stearyl | C_25_H_72_MgM_4_O_4_ | 37.174 | 841.5 | 1000164-49-7 | 4.71 |
|  | Unknown |  | 37.267 |  |  | 9.63 |
|  | Ergosterol | C_28_H_44_O | 37.374 | 396.6 | 57-87-4 | 4.53 |
|  | Bacteriochlorophyll-c-stearyl | C_25_H_72_MgM_4_O_4_ | 37.548 | 841.5 | 1000164-49-7 | 4.41 |
|  | Ergosterol | C_28_H_44_O | 37.755 | 396.6 | 57-87-4 | 29.23 |
|  | Campesterol | C_28_H_44_O | 38.010 | 400.7 | 474-62-4 | 13.15 |
|  | Tetracosamethyl-cyclododecasiloxane | C_24_H_72_O_12_Si_12_ | 38.214 | 889.8 | 18919-94-3 | 32.54 |
|  | Stigmasterol | C_29_H_48_O | 38.420 | 412.7 | 83-48-7 | 28.36 |
|  | Unknown |  | 38.601 |  |  | 3.00 |
|  | beta-d-Mannofuranose,2,3:5,6-di-O-ethylboranediyl-1-O-(10-undecen-1-yl)- | C_21_H_38_B_2_O_6_ | 38.772 | 408.1 | 1000155-22-1 | 8.15 |
|  | Ethanol, 2-(9-octadecenyloxy)-, (z)- | C_20_H_40_O_2_ | 38.950 | 312.5 | 5353-25-3 | 1.77 |
|  | gamma-Sitosterol | C_29_H_50_O | 39.228 | 414.7 | 83-47-6 | 100.00 |
|  | Stigmasterol | C_29_H_48_O | 39.308 | 416.72 | 19466-47-8 | 17.10 |
|  | Cholest-5-en-3-ol, 24-propylidene-, (3 beta)- | C_30_H_50_O | 39.360 | 426.72 | 56362-29-3 | 12.35 |
|  | Unknown |  | 39.431 |  |  | 1.27 |
|  | Unknown |  | 39.518 |  |  | 9.56 |
|  | Testosterone propionate | C_22_H_32_O_3_ | 39.635 | 344.5 | 57-85-2 | 11.20 |
|  | Tetracosamethyl-cyclododecasiloxane | C_24_H_72_O_12_Si_12_ | 39.738 | 889.8 | 18919-94-3 | 39.86 |
|  | Tris(tert-butyldimethylsilyloxy) arsane | C_18_H_45_AsO_3_Si_3_ | 39.886 | 468.7 | 1000366-57-5 | 6.25 |
|  | 4,22-Stigmastadiene-3-one | C_29_H_46_O | 39.990 | 410.7 | 20817-72-5 | 18.54 |
|  | 4-n-Hexylthiane, S, S-dioxide | C_11_H_22_O_2_S | 40.090 | 218.36 | 70928-52-8 | 7.89 |
|  | Tris(tert-butyldimethylsilyloxy) arsane | C_18_H_45_AsO_3_Si_3_ | 40.226 | 468.7 | 1000366-57-5 | 6.04 |
|  | Tris(tert-butyldimethylsilyloxy) arsane | C_18_H_45_AsO_3_Si_3_ | 40.342 | 468.7 | 1000366-57-5 | 1.145 |
